# Evidence of the correlation between air pollution and different types of birth defects: based on a distribution-lag non-linear model

**DOI:** 10.3389/fpubh.2025.1562461

**Published:** 2025-04-09

**Authors:** Wen Sun, Qingqing Dong, Yingying Zhang, Hui Wang, Youqiang Wang, Wenjie Yuan, Leyao Wang, Xianhong Shi, Yuhong Feng, Haiwei Wang, Xiaodan Wang, Yingbin Ren, Lihong Wang, Lijian Lei, Wenxia Song

**Affiliations:** ^1^Department of Epidemiology, School of Public Health, Shanxi Medical University, Shanxi Key Laboratory of Environmental Health Impairment and Prevention, NHC Key Laboratory of Pneumoconiosis, MOE Key Laboratory of Coal Environmental, Taiyuan, Shanxi, China; ^2^Changzhi Maternal and Child Health Care Hospital, Changzhi City Key Laboratory of Birth Defect Prevention and Control, Changzhi, Shanxi, China

**Keywords:** air pollution, BDs, distribution lag non-linear model, lag, expose

## Abstract

**Introduction:**

As global fertility rates decline, exploring the root causes of birth defects (BDs) becomes urgent. Air pollution, with its ability to penetrate the placental barrier as exogenous toxins, has garnered notable attention in this regard.

**Methods:**

BD data was collected from five hospitals in Changzhi City birth from 2019 to 2021, air quality data originated from hourly observations at five monitoring stations within the city. Using the distributed lag non-linear model (DLNM), the study aimed to determine the non-linear exposure-lag-effect relationship, evaluating the delayed impact of weekly air pollution on fetal BD risk. During the period under study, the prevalence of BDs was 19.95‰.

**Results:**

Our findings indicate that exposure to air pollutants during early and mid-pregnancy elevated the risk of BDs. Specifically, for each 10 μg/m^3^ increase of SO_2_, NO_2_, PM_10_, PM_2.5_, O_3_, and CO, the risk of congenital heart defects (CHDs) increased. Peaking at specific gestational weeks: SO_2_ at week 17, NO_2_ at week 23, PM_10_ at week 21, PM_2.5_ at week 16, O_3_ at week 8, and CO at week 40. Additionally, a rise of 10 μg/m^3^ in PM_10_ during weeks 4–10 of gestation significantly elevated the risk of polydactyly, peaking at week 6. Increases in PM2.5 and CO were associated with an elevated risk of external ear malformations, peaking at week 18 and week 19, respectively. Furthermore, higher concentrations of NO_X_ and NO increased the risk of syndactyly, peaking at week 0 for both pollutants. Finally, increments of 10 μg/m^3^ in NO_2_, NO_X_, NO, and PM_10_ were all significantly associated with an increased risk of cleft lip and/or palate, peaking at week 3 for NO_2_, NO_X_, NO, and PM_10_. Exposure to air pollutants elevates BD risk, with critical periods during the first and second trimesters. The association between different pollutants and the classification of BDs also varies.

**Discussion:**

Exposure to pollutants during pregnancy increases the risk of birth defects in newborns, especially SO_2_, PM_10_, PM_2.5_ and O_3_. In light of these findings, we recommend that, while overall regional air quality improvements remain essential, specific targeted measures should be implemented for pregnant women, who represent a particularly vulnerable population. These targeted recommendations not only aim to reduce exposure risks for pregnant women and their fetuses but also offer practical insights for public health policy and interventions.

## Introduction

Birth defects (BDs), commonly referred to as congenital malformations, encompass a range of structural, functional, and metabolic disorders that manifest during fetal development. These include conditions such as congenital heart diseases (CHDs), neural tube defects (NTDs), cleft lip, limb reduction defects, and Down syndrome (1, 2). Worldwide, BDs have an estimated overall prevalence of 46.5‰ (53), with an annual incidence of 7.05‰ in the United States (3) and ranging from 7.15‰ to 191.84‰ in China, resulting in approximately 900,000 new cases annually (4–6). These defects are a leading cause of early abortion, stillbirths, neonatal deaths, and infant deaths, significantly impacting population birth quality and posing a critical public health challenge to population sustainability.

Congenital heart diseases stand as the most frequently occurring BDs globally (56), leading to a significant number of infant deaths. With a prevalence rate of 684 per 10,000 worldwide (57), China alone witnesses around 100 cases per 10,000 births annually (7). Surgical procedures cater to about 70,000 CHD patients each year. NTDs follow closely in incidence rates, with a global prevalence of 1.0 ~ 10.0 per 1,000 births (8). These birth defects not only impose substantial medical expenses but also elevate the risk of prenatal and infant mortality, in addition to fostering life-long disabilities. Addressing these challenges calls for heightened awareness and improved healthcare resources.

Regarding the etiology of BDs, in recent years, many scholars have paid attention to the exposure of environmental factors, apart from genetic factors. For developing countries similar to China, air pollutants such as PM_2.5_, SO_2_, NOx, etc. are considered major risk factors for numerous diseases and have become a global environmental issue. Epidemiological studies suggest that long-term or short-term exposure to air pollutants may elevate risks of pregnancy complications, premature births, low birth weight, and fetal malformations (9, 10, 43, 54). For instance, a study conducted in Wuhan city, covering 70,854 singletons from 2013 to 2018, revealed that early pregnancy exposure to air pollutants increases the risk of BDs (11). Concurrently, another survey encompassing 1,434,998 live births across 30 provinces in China showed that with a 10 μg/m^3^ increase in PM_2.5_ concentration 3 months before pregnancy, the risk of BDs was 1.02 (95% CI:1.00–1.05) (12). When it comes to specific CHD subtypes, the impact of various pollutants like PM_10_ and SO_2_ remains inconclusive, with varying interpretations in the research (13, 14). Air pollution has been shown to have widespread and profound effects on the health of pregnant women and their fetuses, especially in the context of early pregnancy exposure. Epidemiological studies have indicated that prenatal exposure to air pollution may increase the risk of congenital heart defects (CHDs). A recent meta-analysis demonstrated a significant association between PM_2.5_, PM_10_, NO_2_, CO, and O_3_ and specific CHD subtypes (14). A case–control study conducted across nine U.S. states found that NO_2_ exposure was positively associated with coarctation of the aorta (COA) and pulmonary valve stenosis (PVS), while PM_2.5_ exposure was positively correlated with hypoplastic left heart syndrome (HLHS) but negatively associated with atrial septal defects (ASD) (15). Additionally, studies have reported that exposure to O_3_ and PM_10_ during the first trimester of pregnancy increases the risk of ventricular septal defects (VSD), atrial septal defects (ASD), and patent ductus arteriosus (PDA), whereas the associations between SO_2_, NO_2_, CO, and CHDs were weak or negative (16). However, although the existing literature has extensively explored the effects of air pollution on pregnancy outcomes, studies of the non-linear association and lag effects between air pollution and subtypes of BDs remain relatively scarce, especially in developing countries such as China. Based on these deletions, the non-linear model of distribution lag (DLNM) was used to systematically evaluate the lag effect of air pollution on different types of BDs.

Northern China, home to numerous heavy industrial zones, has been facing severe coal-based air pollution and frequent haze events. Changzhi City in Shanxi Province, known for its coal mines and heavy industries, experiences soot-type air pollution during the six-month winter heating period. Investigating the relationship between high-risk pollutants and various subtypes of BDs holds practical importance for setting air quality standards and preventing BDs. Therefore, the purpose of this study is to analyze the correlation between air pollution and various birth defects through more comprehensive data covering all types of birth defects in Changzhi City, and provide a reference for such research.

## Methods

### Data resources

Data from the Maternal and Child Health System of Changzhi City, which comprises five 3A hospitals, provides valuable information on maternal and infant health. The “Birth Defects Registration Card” and “Quarterly Report on Number of Perinatal Births” contain detailed data on maternal status, birth status, birth defect diagnosis, and family history. This data is essential for monitoring and improving maternal and infant health outcomes in Changzhi City.

A comprehensive study was conducted in Changzhi City from January 2019 to December 2021, focusing on pregnant mothers residing in the area. A total of 83,350 infants were included in the research, with 1,663 of them being diagnosed with BDs. The diagnosis of BDs was made using a combination of physical examinations, ultrasonography, X-ray examinations, and genetic diagnostic methods. These diagnostic methods were in line with the codes Q00–Q99 of ICD-10 (17, 18). The inclusion criteria for this study were: mother-infant pairs who conceived and gave birth in Changzhi City, Shanxi Province, between January 2019 and December 2022; permanent residence in Changzhi City during pregnancy, verified by the registered address; recruitment from monitoring hospitals in Changzhi City with complete maternal demographic and medical history collected at hospital admission; gestational age determined based on last menstrual period (LMP) and confirmed via ultrasound before 20 weeks of gestation; and newborns diagnosed with congenital heart disease (CHD) according to the Chinese Maternal and Child Health Monitoring Manual to ensure standardization. Exclusion criteria included: women who were not permanent residents of Changzhi City during pregnancy or had missing residential information; incomplete maternal demographic or medical history records; uncertain gestational age due to missing LMP data or lack of ultrasound confirmation; newborns not diagnosed with CHD based on the Chinese Maternal and Child Health Monitoring Manual; and infants with incomplete birth records, such as missing birth date, gestational age, sex, maternal age, gravidity, or parity information. All of the study participants signed the informed consent form. All of the study participants signed the informed consent form. The study aimed to understand the prevalence and characteristics of BDs among infants in Changzhi City during the specified period, providing valuable insights for healthcare professionals and policymakers in the region.

### Quality controls

To guarantee the report’s accuracy, a physician at each registered hospital was mandated to fill out a quarterly form alongside the BDs Registration Card. Both cards and tables were rigorously reviewed and audited by maternal and child health hospitals and health administrative departments. The monitoring data quality criteria specified a 100% form completion rate, a form item error rate of less than 1%, an input error rate of less than 1%, and a missed BDs rate of less than 1% (19).

### Exposure assessment

The ambient air pollution data utilized in this study originated from the weather information data collected by the Changzhi Environmental Protection Bureau between January 2019 and December 2021. Individual exposure levels were determined using the mean values from the five monitoring points. The monitored pollutants encompassed PM_2.5_, PM_10_, SO_2_, CO, NO_2_, and O_3_. The CO concentration was gauged in mg/m^3^, while the remaining pollutants were measured in μg/m^3^. The daily maximum 8-h average concentration of O_3_ was calculated, while the remaining pollutants were determined based on the 24-h mean values obtained from the five monitoring points.

### Statistical analysis

Given that weekly BDs represent a relatively low-probability event compared to the overall number of births, this study employed the Distributed Lag Non-linear Model (DLNM) based on the Poisson distribution to explore the association between weekly infant BDs and atmospheric pollution levels. Since its introduction by Schwartz J, Poisson regression analysis has become a staple in environmental epidemiology, particularly in assessing the health effects of air pollution (20). The DLNM model was constructed in two stages: initially, a fundamental “cross-basis function” was utilized to generate a two-dimensional matrix representing exposure levels and time, outlining their interrelationship. Subsequently, a regression model incorporating the logarithmic function was established, incorporating covariates and outcome variables, such as the base matrix. The DLNM model provided a “crossbred” predicted function tailored to reflect the association between exposure and outcome.

The research explores the impact of prenatal and preconception exposure to atmospheric pollution on the risk of BDs. The literature shows that air pollution exposure may have a gradually cumulative negative effect on the development of the fetus, and its effects may not appear until a long time in late pregnancy or after childbirth, then birth defects are closely related to exposure throughout pregnancy and even before pregnancy. Therefore, based on biological rationality and the support of previous studies, this study chose a longer hysteresis (40 weeks) to ensure that the long-term impact of air pollution exposure on birth defects can be captured. By analyzing weekly average levels of exposure data with a maximum lag of 40 weeks, the study identifies susceptible windows for BDs. Time variables are included in the model using non-linear smoothing functions to control confounding factors. In model construction, we first compare the fitting of different function forms (linear, polynomial, natural spline) through the AIC criteria. For the exposure dimension, we choose a linear model. For the lag dimension, select the 4th polynomial. This allows for a deeper understanding of the relationship between different exposure levels and BDs. The findings highlight the importance of reducing exposure to pollution during critical periods of pregnancy to reduce the risk of birth defects. The formula used in the analysis is detailed for further reference.


$$\begin{array}{l} \mathrm{YtPoisson}\left(\mu t\right)\\ \log\left(\mu t\right)=\alpha +\beta Xt+\mathrm{ns}\left(\mathrm{time},df\right) \end{array}$$

The formula used for assessing the impact of air pollution on the occurrence of birth defects includes various elements such as gestational week, number of birth defects, regression coefficient for air pollutants, pollutant concentrations, natural smoothing function for non-linear variables, meteorological data, and interception. By utilizing the “cross” function, lag-effect relationship curves are plotted to evaluate how air pollution affects the occurrence of birth defects. This comprehensive method allows for a detailed analysis of the relationship between air pollution and birth defects, providing valuable insights into the potential health risks associated with exposure to pollutants during pregnancy.

SPSS 27.0 software was utilized for an overview and chi-square test, while R 3.4.0 software was employed for DLMN regression analysis. Statistical significance was denoted by *p* < 0.05.

## Results

### Descriptive statistics

Between January 2019 and December 2021, a research study involving 83,350 infants revealed that 1,663 of them had birth defects, resulting in an incidence rate of 19.95‰. Analysis of the data indicated that individuals under 20 years of age (29.41‰), those with low levels of education (27.65‰), and babies born in the spring season (22.36‰) displayed the highest rates of birth defects. Furthermore, male fetuses exhibited a higher prevalence of birth defects compared to female fetuses, with rates of 21.97‰ and 17.83‰ respectively. These findings shed light on the various factors associated with the occurrence of birth defects (Table 1).

**Table 1:** Prevalence of BDs among different participant subgroups.

Variable	Infants	BDs	IR (‰)	*χ* ^2^	*p*-value
Sum	83,350	1,663	19.95		
Maternal age				30.559	<0.001
<20	442	13	29.41		
(20, 25)	12,128	221	18.22		
(25, 30)	38,666	711	18.39		
(30, 35)	24,325	504	20.72		
≥35	7,789	214	27.47		
Maternal education				230.823	<0.001
College or higher	38,414	833	21.68		
Senior high school	24,640	666	27.03		
Junior high school	19,175	133	6.94		
Primary school or lower	1,121	31	27.65		
Season of birth				10.316	0.016
Spring	20,972	469	22.36		
Summer	21,603	431	19.95		
Autumn	20,729	404	19.49		
Winter	20,046	359	17.91		
Child’s sex				17.545	<0.001
Male	42,641	937	21.97		
Female	40,709	726	17.83		
Gestational age				4096.305	<0.001
≥37 weeks	77,979	958	12.29		
28 ~ 36 weeks	4,219	394	93.39		
<28 weeks	1,152	311	269.10		
Birth weight				4457.82	<0.001
2,500 g ~ 4,000 g	73,060	898	12.29		
<2,500 g	3,499	663	189.48		
>4,000 g	6,791	102	15.02		

The seasons of birth were categorized based on month ranges: spring (March–May), summer (June–August), autumn (September–November), and winter (December–February).

The data presented in Table 2 illustrates the average concentrations of various atmospheric pollutants in Changzhi City between 2019 and 2021. The average levels were found to be 15.71 μg/m^3^ for SO_2_, 1.11 mg/m^3^ for CO, 29.61 μg/m^3^ for NO_2_, 76.13 μg/m^3^ for O_3_, 79.45 μg/m^3^ for PM_10_, 43.19 μg/m^3^ for PM_2.5_, 41.35 μg/m^3^ for NO_X_, and 7.64 μg/m^3^ for NO. Notably, the yearly average concentrations of PM_2.5_ and PM_10_ exceeded the second-level limit values set by the national standard “Ambient Air Quality Standard” (GB3095-2012), while the other pollutants remained within the first-level standards.

**Table 2:** Air pollutant distribution in Changzhi from 2019 to 2021.

Index	Mean	SD	Min	*P*_5_	*P*_25_	*P*_50_	*P*_75_	*P*_95_	Max
SO_2_/(μg/m^3^)	15.71	8.79	7.14	8.52	9.85	12.58	17.78	33.35	68.44
CO/(mg/m^3^)	1.11	0.45	−0.55	0.59	0.81	1.04	1.31	2.02	3.75
NO_2_/(μg/m^3^)	29.61	13.53	3.47	11.62	19.11	27.41	34.17	54.72	84.86
O_3_/(μg/m^3^)	76.13	36.05	4.59	24.74	47.92	71.77	100.46	140.94	192.59
PM_10_/(μg/m^3^)	79.45	51.03	4.96	29.53	52.18	69.26	94.08	152.13	836.80
PM_2.5_/(μg/m^3^)	43.19	28.70	0.09	14.28	25.27	35.73	51.67	99.30	292.92
NO_X_/(μg/m^3^)	41.35	27.48	4.41	13.22	21.93	32.98	53.31	98.10	190.70
NO/(μg/m^3^)	7.64	10.26	−0.03	0.71	1.42	3.15	9.48	29.75	69.01

**Figure 1:**
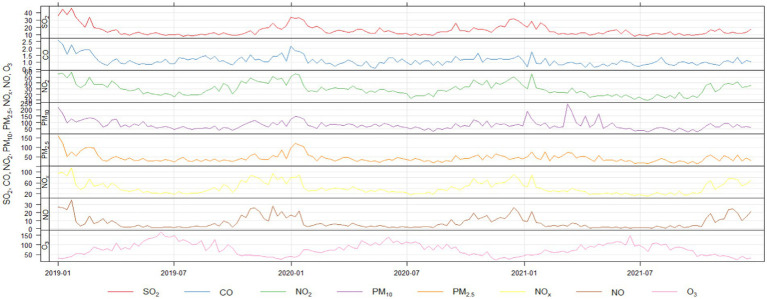
The concentrations of air pollutants fluctuated over days.

Throughout the period analyzed, daily average concentrations of these pollutants occasionally surpassed the national first-level standards and even breached the second-level benchmarks, as depicted in Figure 1. Measures must be taken to address these concerning levels to safeguard public health and improve overall air quality in the region.

### The correlation among atmospheric pollutants

**Figure 2:**
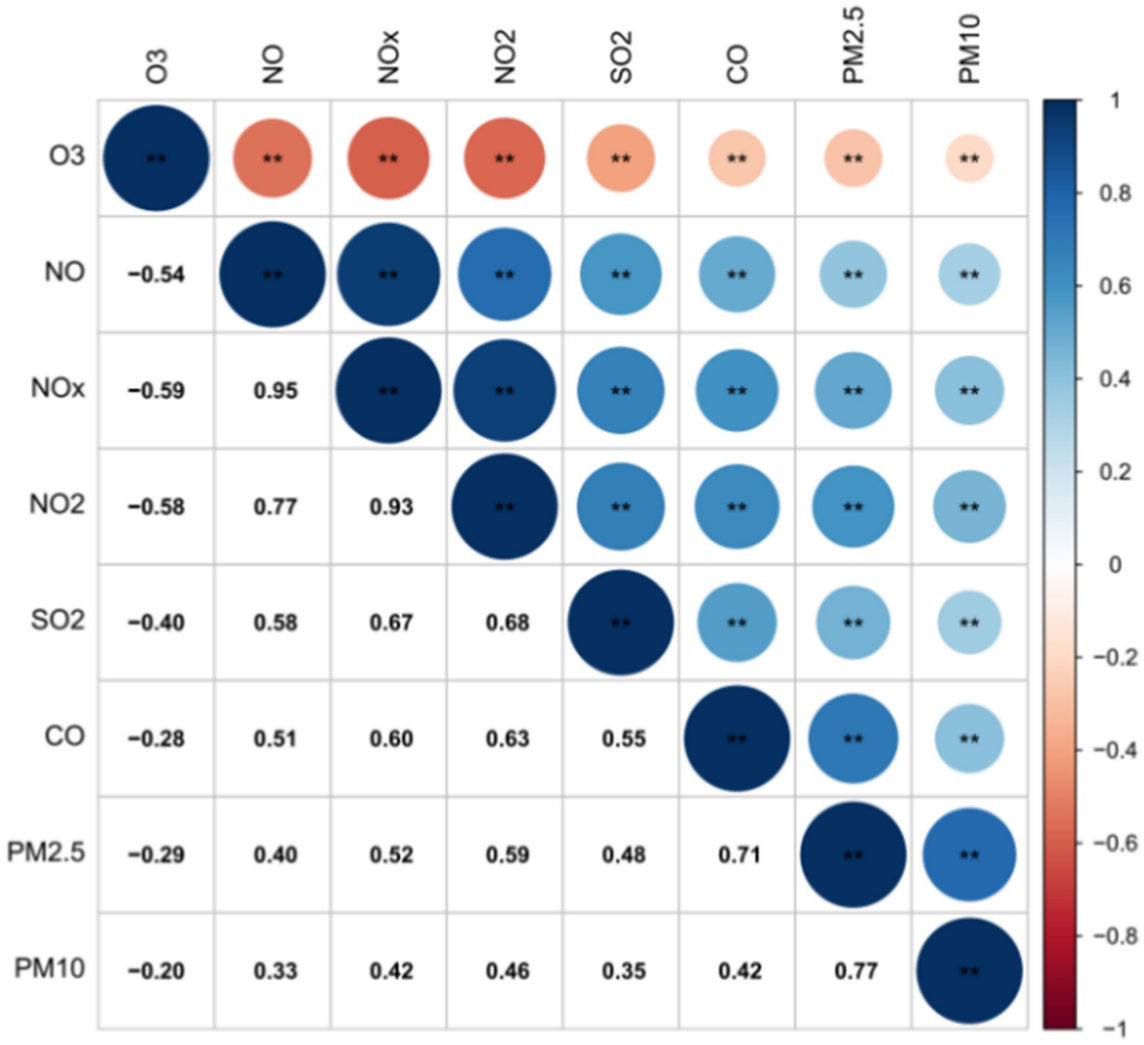
Significant correlation among atmospheric pollutants. ^**^ means at the 0.01 level (two-tailed), the correlation was significant.

Positive correlations were observed among all pollutants except for O_3_. The strongest relationships were found between NO_X_ and NO, as well as NO_2_. Additionally, PM_2.5_ showed strong connections with PM_10_ and CO. Conversely, O_3_ displayed weaker correlations with PM_10_, PM_2.5_, CO, and SO_2_. PM_10_ had weaker links with NO and SO_2_, while PM_2.5_ had a relatively weaker relationship with NO. The data in Figure 2 illustrates these correlations, emphasizing the interconnected nature of air pollutants and their impact on air quality.

### Associations of weekly air pollutants exposure with BDs

**Figure 3:**
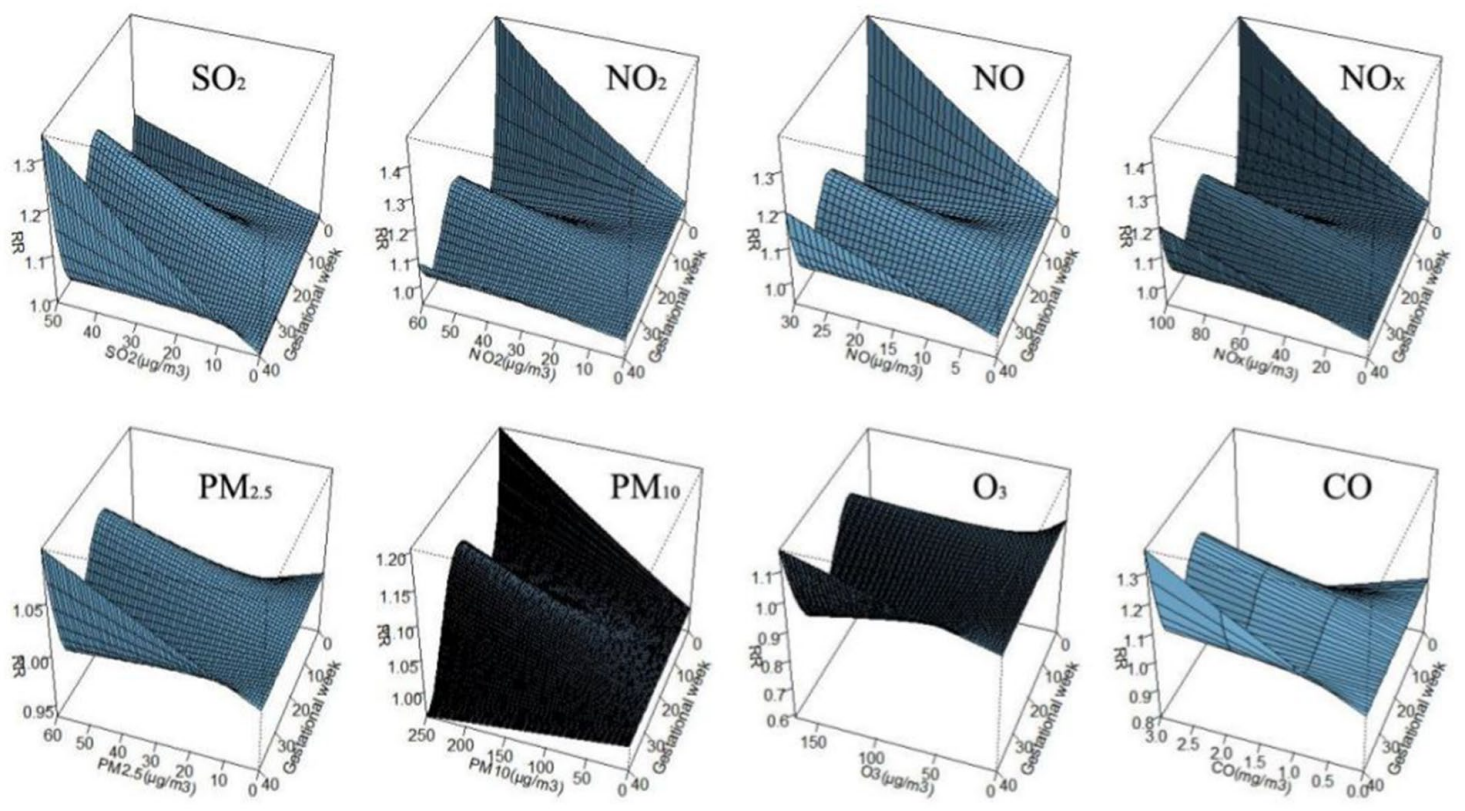
The effect of air pollutant exposure on BDs at different concentrations and gestational weeks.

Figure 3 shows the correlation between exposure to air pollutants and adverse BDs is clearly illustrated, which displays the impact of different pollutant concentrations during various gestational weeks. As pollutant concentrations increase, the relative risk (RR) values also rise, indicating a higher likelihood of negative effects on birth outcomes. However, the specific sensitive windows for each pollutant vary. NO_2_, NO_X_, NO, and PM_10_ exhibit peak RR values during early and late pregnancy, whereas PM_2.5_, CO, and SO_2_ show the opposite trend. Interestingly, O_3_ shows a U-shaped curve in RR values. These findings emphasize the importance of understanding the timing and impact of air pollutant exposure during pregnancy to mitigate potential risks to maternal and fetal health.

**Figure 4:**
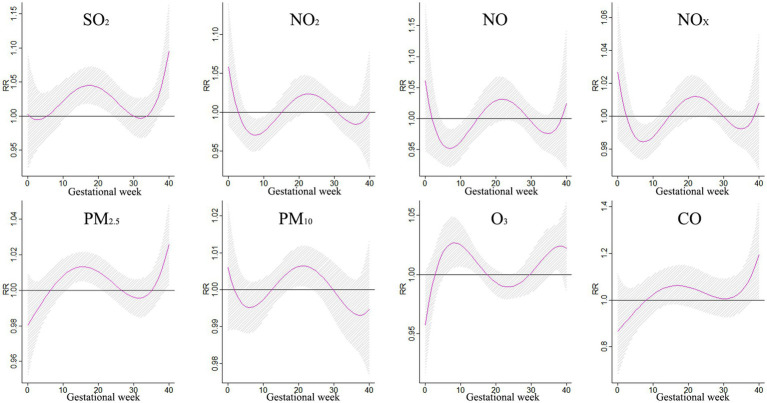
The impact of a 10 μg/m^3^ rise in individual pollutant exposure on BDs.

The impact of air pollution on BDs during different gestational periods was examined in a study, with findings presented in Figure 4. Exposure to SO_2_ was found to increase the risk of BDs between weeks 12–26, with the greatest impact observed at week 19. NO_2_ showed a significant impact on BDs at weeks 0–1 and 10–29, reaching its peak at week 0. Similarly, NO_X_ and NO also exhibited significant impacts on BDs during specific gestational periods. Exposure to particulate matter such as PM_10_ and PM_2.5_ increased the risk of BDs, with the highest impact observed at week 22. These findings highlight the importance of reducing exposure to air pollutants during pregnancy to safeguard the health of unborn babies.

### Associations of weekly air pollutants with CHDs

Between 2019 and 2021, the most frequently reported birth defects were congenital heart defects, polydactyly, external ear malformations, syndactyly, and cleft lip/palate. This study explores the potential impact of weekly air pollution exposure on the occurrence of these specific birth defects.

**Figure 5:**
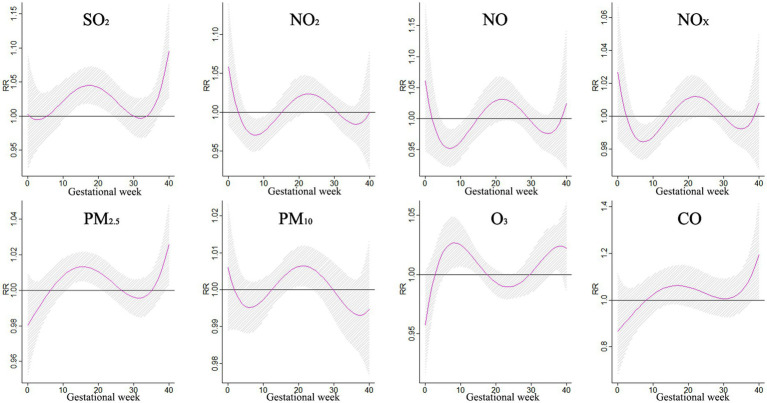
The effect of a 10 μg/m^3^ increase in single pollutant exposure on CHD.

The findings from Figure 5 shed light on the association between exposure to various air pollutants and the risk of CHDs at different stages of pregnancy. It was discovered that exposure to SO_2_ during weeks 12–24 of gestation increased the risk of CHDs, with the relative risk (RR) ranging from 1.027 to 1.045. The most significant impact was observed during week 17, with an RR of 1.045 and a 95% confidence interval (CI) of 1.018–1.072. Similarly, NO_2_ exposure was found to have a significant effect on CHDs during lag 6–11 weeks and lag 23–26 weeks, peaking at lag 23 weeks with an RR of 1.024 and a 95% CI of 1.001–1.047. In contrast, exposure to PM_10_ during weeks 18–24 increased the risk of CHDs, with the RR ranging from 1.005 to 1.006. The strongest effect was observed during week 21, with an RR of 1.006 and a 95% CI of 1.001–1.012. Moreover, exposure to PM_2.5_ during weeks 11–21 was also associated with an elevated risk of CHDs, with the RR ranging from 1.009 to 1.013. The most significant impact occurred during week 16, with an RR of 1.013 and a 95% CI:1.005–1.021. Additionally, O_3_ exposure during lag 6–14 and lag 36–38 weeks had a notable effect on CHDs, peaking at lag 8 weeks with an RR of 1.027 and a 95% CI of 1.005–1.049. Furthermore, exposure to CO during weeks 39–40 significantly increased the risk of CHDs, with the RR ranging from 1.147 to 1.194. The most pronounced effect was observed during week 40, with an RR of 1.194 and a 95% CI: 1.006–1.418. These findings underscore the importance of reducing exposure to these air pollutants during pregnancy to mitigate the risk of CHDs.

### Associations of weekly air pollutants with polydactyly

**Figure 6:**
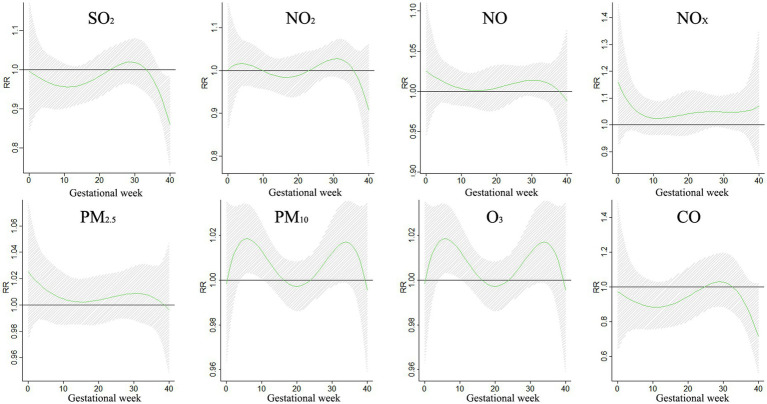
The effect of a 10 μg/m^3^ increase in single pollutant exposure on polydactyly.

The illustration in Figure 6 shows the influence of a 10 μg/m^3^ rise in pollution on the occurrence of polydactyly during various stages of pregnancy. Between weeks 4–10, exposure to PM_10_ was associated with a higher risk of polydactyly, with the relative risk ranging from 1.013 to 1.019. The most significant impact was observed in week 6, with a relative risk of 1.019 (95% CI: 1.003–1.034).

### Associations of weekly air pollutants with external ear malformations

**Figure 7:**
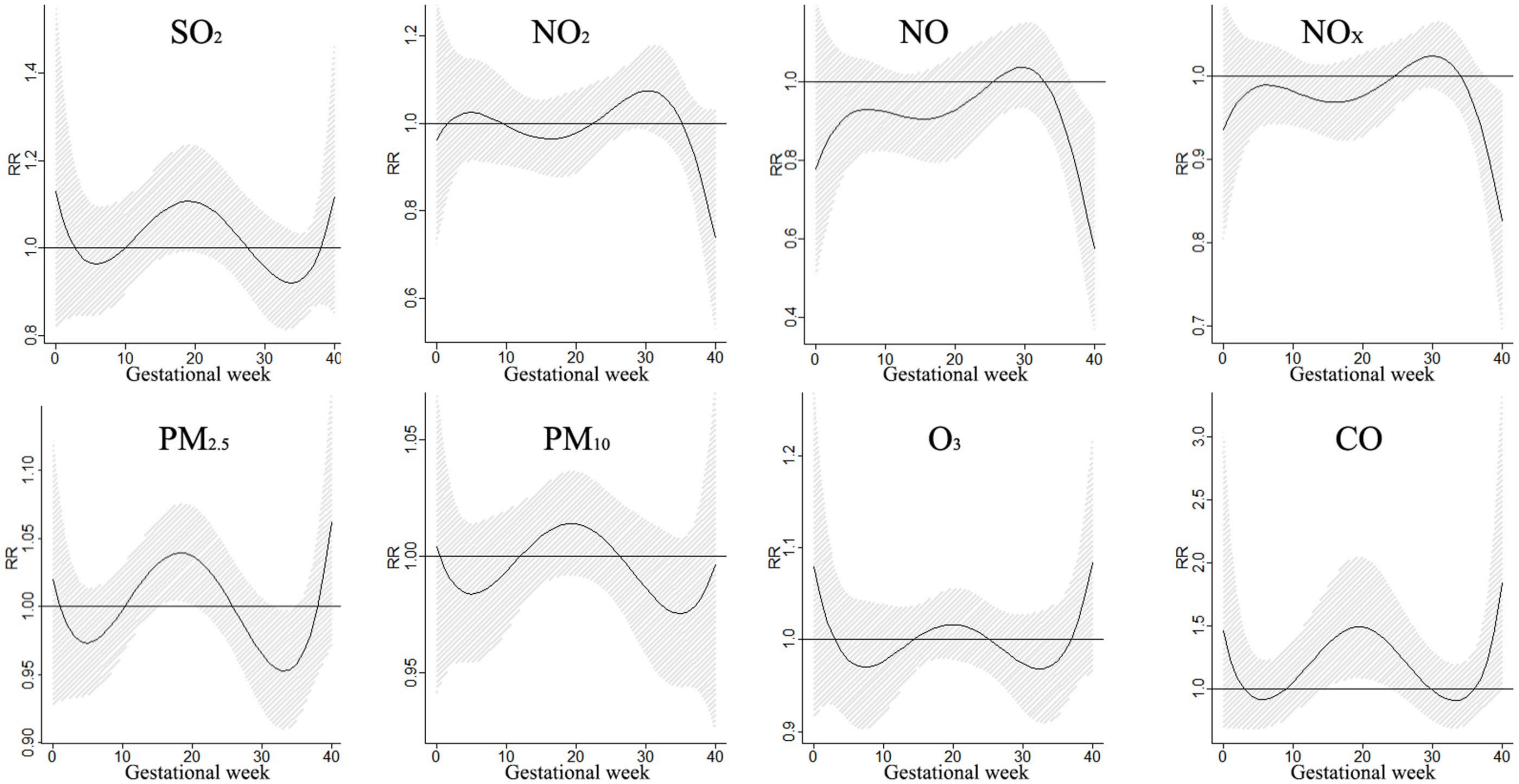
The effects of a 10 μg/m^3^ increase in single pollutant exposure on external ear malformations.

The impact of air pollution on external ear malformations during pregnancy was investigated in a study that focused on the effects of PM_2.5_ and CO exposure. Results showed that a 10 μg/m^3^ increase in PM_2.5_ levels led to a significant risk of external ear malformations during gestational periods between 16–20 weeks and 32–34 weeks, peaking at 18 weeks. On the other hand, exposure to CO during weeks 15–24 increased the risk of external ear malformations, with the highest risk observed at week 19. These findings shed light on the potential health risks associated with exposure to specific pollutants during pregnancy, emphasizing the importance of monitoring pollutant levels at different stages of gestation. Understanding these risks can support the development of targeted interventions to protect the health of both mothers and fetuses (Figure 7).

### Associations of weekly exposure with air pollution on syndactyly

**Figure 8:**
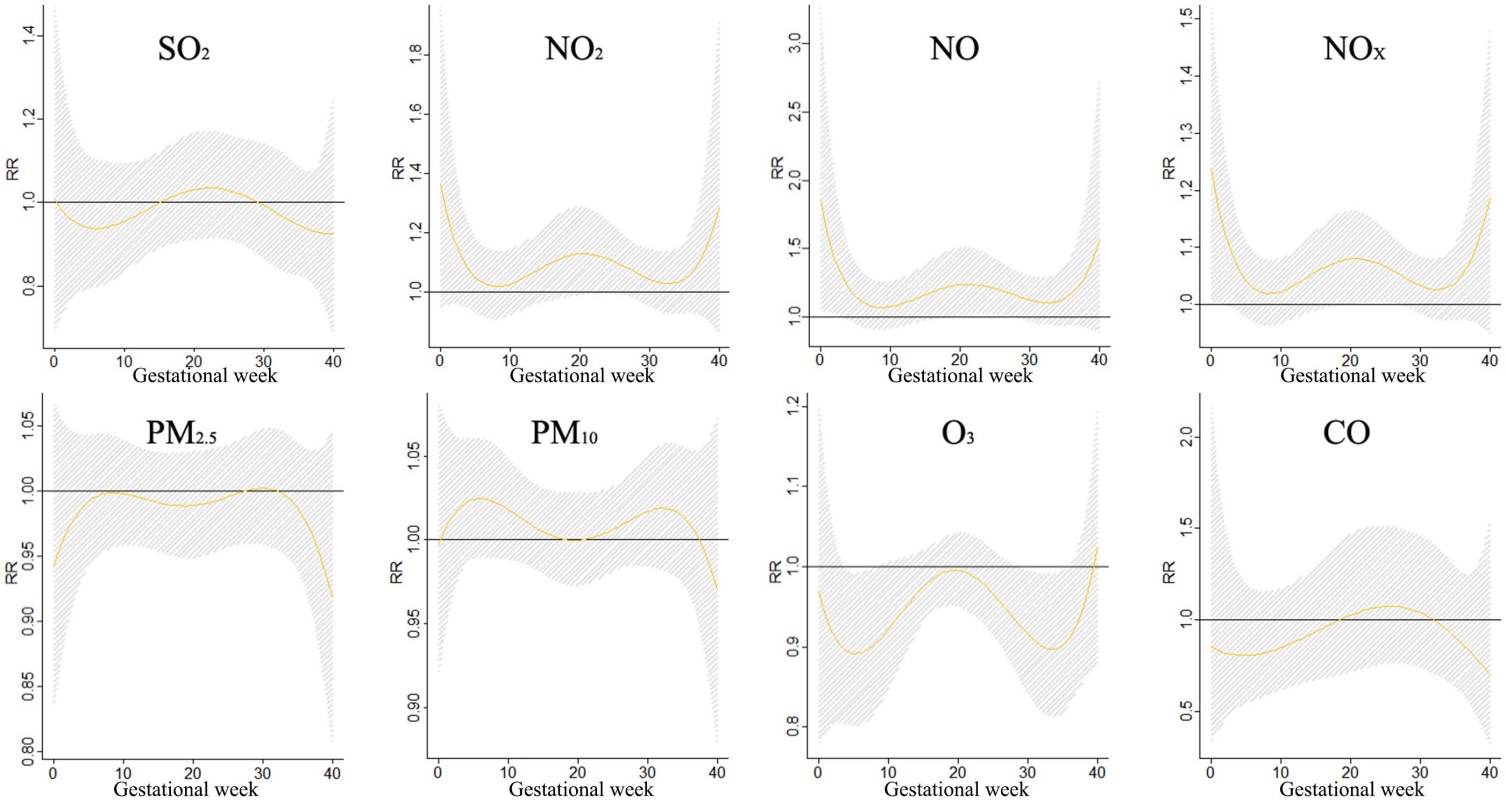
The effects of a 10 μg/m^3^ increase in single pollutant exposure on syndactyly.

The impact of a 10 μg/m^3^ increase in single pollutant exposure on syndactyly during different gestational periods is demonstrated in Figure 8. The study revealed that NO_X_ had a significant impact on syndactyly at lag 0–2 weeks and lag 20–27 weeks, with the highest risk occurring at lag 0 weeks (RR = 1.237, 95%CI:1.003–1.525). Similarly, NO exposure showed a significant impact at lag 0–3 weeks and lag 18–28 weeks, peaking at lag 0 weeks (RR = 1.857, 95%CI: 1.050–3.283). These findings offer valuable insights into how specific pollutants can influence syndactyly development during pregnancy. It underscores the importance of considering pollutant exposure levels at different gestational stages for a more thorough risk assessment and targeted interventions to protect the health of both mother and fetus.

### Associations of weekly air pollutants exposure with cleft lip and/or cleft palate

**Figure 9:**
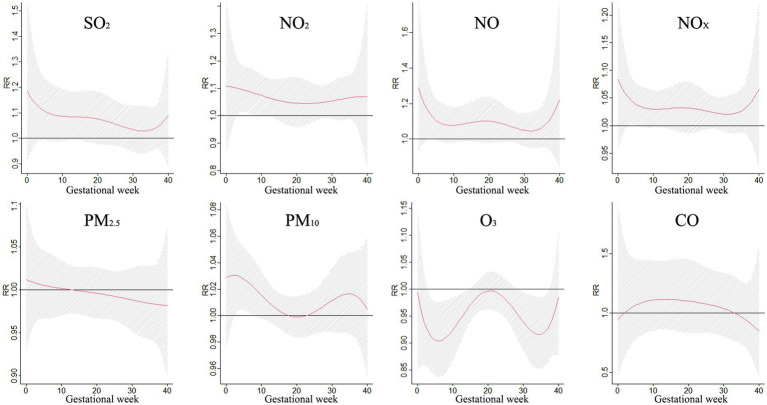
The effects of a 10 μg/m^3^ increase in single pollutant exposure on cleft lip and/or cleft palate.

The impact of exposure to various pollutants on the risk of cleft lip and/or cleft palate during pregnancy was analyzed in Figure 9. A 10 μg/m^3^ increase in single pollutant exposure was found to have differing effects depending on the gestational period. For example, exposure to NO_2_ from weeks 3 to 12 increased the risk of cleft lip and/or cleft palate, with the risk ratio (RR) ranging from 1.066 to 1.101. The highest risk was observed in the 3rd week of gestation, with a RR of 1.010 (95% CI: 1.003–1.209). Similarly, exposure to NO_X_ from weeks 3 to 5 increased the risk, with the RR ranging from 1.039 to 1.051, and the strongest effect seen in the 3rd week (RR = 1.051, 95%CI: 1.002–1.103). Moreover, exposure to NO from weeks 3–4 increased the risk, with the RR ranging from 1.124 to 1.150, and the highest effect was observed in the 3rd week (RR = 1.150, 95%CI: 1.006–1.316). Additionally, exposure to PM_10_ from weeks 3–8 increased the risk, with the RR ranging from 1.021 to 1.030. The peak effect was seen in the 3rd week (RR = 1.030, 95% CI: 1.003–1.058). These findings shed light on the potential impact of pollutant exposure on the development of cleft lip and/or cleft palate in pregnant individuals. Understanding these effects at different gestational periods is crucial for effective risk assessment and targeted interventions to safeguard maternal and fetal health.

## Discussion

The prevalence of BDs in Changzhi from January 2019 to December 2021 was 19.95‰, with 1,663 cases among 83,350 infants. This rate is comparable to the global average of 2–4% (21) and similar to findings in Wuhan (19.08‰) (11) but higher than Guilin (13.55‰) (22), despite the latter having better air quality (23, 24). BDs, particularly CHDs, impact millions of newborns annually worldwide. Various factors such as gestational age, maternal education, season of birth, child’s sex, gestational age, and birth weight were identified as potential risk factors for BDs in this study. We found similarities with research in Hunan (25), showing a higher occurrence of BDs in males compared to females. Additionally, a study by Xie et al. (26) noted a higher prevalence of congenital heart defects in male infants, suggesting that boys may be more vulnerable to the effects of air pollution than girls due to differences in autosomal methylation patterns between the sexes (27). These findings underline the importance of understanding and addressing the various risk factors associated with BDs to improve prevention and management strategies for affected infants.

The study results highlighted the severe problem of PM pollution in the city, with coal combustion, industrial emissions, and vehicular traffic being identified as the main contributors (28). Contrary to previous research by Zhang et al. (20), our findings suggested that babies born in the spring may face higher risks of birth defects. The seasonal pattern of conception in autumn and winter, characterized by lower temperatures and stagnant air, may lead to the accumulation of PM_2.5_ particles, subsequently increasing their concentration. This heightened exposure to ambient pollutants during pregnancy could potentially elevate the likelihood of birth defects (9). This research underscores the importance of addressing air pollution as a public health concern.

Our research delved into the correlation between air pollution levels and birth defects in Changzhi utilizing a time-series methodology. Employing a Poisson regression model coupled with a DLNM allowed us to comprehensively examine the impact of air contaminants on BDs over an extended period. The outcomes of our study indicated that maternal exposure to various ambient air pollutants such as SO_2_, NO_2_, NO_x_, NO, O_3_, PM_10_, PM_2.5_, and CO significantly elevated the likelihood of birth defects. Further analysis of the single-pollutant model revealed that even a minor increment of 10 units in pollutant concentrations could lead to adverse effects, with the most significant impact observed during weeks 10–28 of pregnancy. Particularly, exposure to PM_10_ or PM_2.5_ during the first trimester heightened the risk of birth defects (29), possibly due to oxidative inflammation, DNA methylation, and endocrine dysregulation in the placenta (30). Notably, previous studies have demonstrated a positive correlation between SO_2_ levels and the risk of birth defects (31, 32). Echoing these findings, investigations in Haikou City underscored that exposure to PM_10_ and SO_2_ in the second and third months of pregnancy could potentially increase the likelihood of birth defects, though no significant effects were observed for NO (33). Additionally, Sun et al.’s (34) study highlighted the significant impact of exposure to PM_2.5_ and SO_2_ during specific gestational weeks, emphasizing the critical periods of vulnerability during the 4th to 13th weeks for PM_2.5_, the 4th to 14th weeks for PM_10_, the 4th to 12th weeks for NO_2_, and the 26th to 35th weeks for O_3_. These findings underscore the importance of considering the timing and type of air pollutants in assessing the risk of birth defects associated with maternal exposure.

Prenatal exposure to air pollution has been linked to an increased risk of CHD. Research has shown that exposure to various pollutants such as O_3_, SO_2_, NO, NO_X_, NO_2_, PM_10_, and PM_2.5_ weekly can significantly increase the likelihood of developing CHDs, with the most pronounced effects seen between the 8th and 23rd weeks of pregnancy. Studies have indicated that exposure to PM_2.5_ during the first trimester of pregnancy can elevate the risk of CHDs (35), while other research conducted in Wuhan observed a strong positive relationship between PM_2.5_ exposure and CHDs during the second and third months of pregnancy (36). Interestingly, a separate study found that PM_2.5_ exposure increased the risk of CHDs specifically between weeks 11 and 21 of pregnancy. Furthermore, research conducted in Fuzhou revealed that PM_10_ was associated with overall fetal cardiovascular malformations, with the most significant effects observed in the second quartile (37). Additionally, adverse associations between CHDs and exposure to SO_2_, NO_2_, NO_X_, NO, and O_3_ have also been documented, highlighting the detrimental impact of various pollutants on fetal heart development. A meta-analysis emphasized the significant impact of CO and SO_2_ on CHDs (38), while a birth cohort study conducted in Foshan City demonstrated that exposure to NO_2_ and O_3_ could increase the incidence of CHDs (39). Conversely, a multi-city cross-sectional study in eastern China found a statistical effect of CO on CHDs (40), differing from findings related to NO_2_ and SO_2_ exposure (41). Studies have also linked maternal exposure to NO_2_ and SO_2_ with CHDs, while in Australia, exposure to O_3_ was associated with an increased risk of aortic artery and valve defects (42). Our study found a significant association between CO exposure and congenital heart defects, which is consistent with existing literature. For example, the high affinity of CO for hemoglobin leads to the formation of carboxyhemoglobin, thereby reducing the blood’s oxygen-carrying capacity (41). This reduction results in inadequate oxygen delivery from the placenta and subsequent fetal hypoxia, which can interfere with the normal proliferation and differentiation of cardiac cells during development. A separate line of evidence, provided data showing that fetal hypoxia can activate oxygen-sensitive transcription factors, such as HIF-1α, which in turn alter the expression of critical growth factors like VEGF and FGF (43). This alteration affects cellular migration and apoptosis during cardiac morphogenesis, thereby increasing the risk of congenital heart defects. These mechanistic insights not only offer a biological explanation for the statistical association observed between CO exposure and congenital heart defects, but they also establish a solid theoretical basis for our future studies aimed at elucidating the underlying biological mechanisms through targeted experiments. The underlying mechanisms behind these associations are thought to involve oxidative stress, inflammation, cellular dysfunction, altered regulatory networks, and epigenetic changes, ultimately leading to adverse structural and functional changes in the cardiovascular system. It is hypothesized that placental or systemic oxidative stress and intrauterine inflammation from air pollution can limit fetal growth and development (44). The strong correlation between premature birth and birth defects in this cohort (*p* < 0.001) suggests that there may be a two-way effect: on the one hand, congenital abnormalities may force the fetus to be delivered early; on the other hand, the exposure of immature organs caused by premature birth to an extrauterine inflammatory environment may aggravate the defect phenotype. This requires further exploration and research in the future (45–48). In conclusion, the detrimental effects of air pollution on the development of CHDs underscore the importance of addressing and reducing exposure to harmful pollutants during pregnancy to minimize the risk of congenital heart defects (49, 50).

Exposure to various air pollutants has been found to significantly increase the risk of certain congenital malformations in newborns. Studies have shown that exposure to sulfur dioxide (SO_2_), particulate matter of various sizes (PM_10_, PM_2.5_), ozone (O_3_), carbon monoxide (CO), nitrogen oxide (NO_X_), and nitrogen dioxide (NO) can all impact fetal development. For instance, exposure to SO_2_, PM_10_, PM_2.5_, O_3_, and CO has been linked to an increased risk of polydactyly, while exposure to NOX, NO, PM_10_, PM_2.5_, and CO has been associated with malformations of the external ear. Additionally, exposure to PM_10_, PM_2.5_, and O_3_ has been shown to increase the risk of syndactyly, and NO_2_, NO_X_, PM_10_, PM_2.5_, and O_3_ have been linked to cleft lip and/or cleft palate. Research by Zhu et al. further supports these findings, with PM_10_, CO, SO_2_, NO_X_, and PM_2.5_ being associated with cleft lip and/or cleft palate (32). PM_2.5_ was negatively correlated with traffic density and cleft lip with or without palate (55). NO_2_ increased risk of congenital polydactyly, cleft palate, and PM_10_ was associated with cleft lip with or without cleft palate (51), preconception and first-trimester PM_10_ exposures are increased risks of polydactyly and syndactyly (52). In addition, maternal exposure to SO_2_, NO_2_, PM and O_3_ is positively associated with the risk of birth defects(34). Interestingly, certain pollutants have been found to have varying impacts depending on timing and external factors. For example, PM_2.5_ was negatively correlated with traffic density and certain pollutants may be influenced by biological mechanisms or protective factors that mitigate their negative effects (55). Factors such as time spent outdoors, commuting time, and lifestyle choices can also play a role in an individual’s actual exposure to ambient air pollution. Pregnant women must be aware of the potential risks associated with air pollution and take precautions to minimize their exposure. Further research is needed to better understand the complex interactions between air pollutants and fetal development to protect the health of future generations.

Our study results showed variations from previous research, attributed to differences in study populations, air pollution levels, exposure time scales, and the magnitude of changes in pollutant concentrations. While previous studies have explored similar topics, our focus on assessing the risk of birth defects for every 10 μg/m^3^ increase in air pollutants provides a unique perspective. These factors highlight the complexities of studying the impact of air pollution on birth defects and reinforce the importance of considering various variables when analyzing such associations.

Research indicates that pregnant women exposed to air pollutants, specifically PM, face a heightened risk of BDs. Additionally, there is a higher likelihood of BDs in women who conceive during the colder months and are exposed to elevated levels of air pollution. This study emphasizes the importance of monitoring air quality for maternal and fetal health.

Our research shows the prevalence of birth defects, and particulate matter is identified as the main air pollutant. Exposure to pollutants during pregnancy increases the risk of birth defects in newborns, especially SO_2_, PM_10_, PM_2.5_ and O_3_. In light of these findings, we recommend that, while overall regional air quality improvements remain essential, specific targeted measures should be implemented for pregnant women, who represent a particularly vulnerable population. For instance, pregnant women should be encouraged to enhance their self-protection awareness by wearing certified masks during periods of poor air quality and minimizing outdoor activities when pollutant levels are high. Additionally, improving indoor air quality through the use of air purifiers and ensuring proper ventilation can further reduce exposure. Monitoring real-time air quality indices can also help in planning daily activities to avoid high-pollution environments. These targeted recommendations not only aim to reduce exposure risks for pregnant women and their fetuses but also offer practical insights for public health policy and interventions.

### Limitations

Our study delved into the correlation between exposure to air pollutants and the occurrence of birth defects, a topic not thoroughly explored in previous research. While our findings shed light on this important issue, it’s crucial to acknowledge the limitations of our study. Firstly, our monitoring period only extended from 28 weeks gestation to 7 days post-delivery, preventing us from examining the impact of air pollution on infants with birth defects beyond this timeframe. Additionally, we generalized the concentrations of air pollutants from monitoring sites across a wide area for all pregnant women, rather than assessing each individual’s exposure during pregnancy. The individual exposure levels of the study subjects were estimated based on the average concentrations from five monitoring stations. This approach characterizes city-wide air pollution exposure rather than precise individual residential or activity-based exposure. While it is true that exposure levels among individuals in the same city and time period may show some degree of similarity, variations still exist due to differences in the distribution of pollution source distribution, meteorological conditions, individual activity patterns, and pregnancy time. In addition, some potential mixed variables may affect the results, such as maternal health conditions such as diabetes or hypertension, occupational exposures, access to prenatal care, temperature and aeroallergenes. Furthermore, factors such as alcohol, tobacco, and drug consumption, occupational hazards, intrauterine conditions, and nutritional intake could all potentially contribute to birth defects, but these were not accounted for in our analysis. It is important to consider these limitations when interpreting the results of our study. Although this study mainly focused on external air pollution exposure, there are a lot of literature studies the relationship between indoor exposure, second-hand smoke exposure and congenital malformation, but the specific quantitative data of indoor air quality and second-hand smoke exposure is not complete, it also suggests that future research can be considered through more accurate indoor air pollution monitoring means to further reduce the potential confounding effects.

## Conclusion

Research conducted in Changzhi city revealed a concerning prevalence of birth defects, with particulate matter being identified as the primary air pollutant. The study noted that exposure to pollutants during the second trimester heightened the risk of neonatal birth defects, particularly SO_2_, PM_10_, PM_2.5_, and O_3_. Factors such as the baby’s gender and the season of pregnancy were found to influence this effect. Environmental agencies must continue their efforts in enhancing air quality to safeguard the well-being of both mothers and their newborns.

## Data Availability

The raw data supporting the conclusions of this article will be made available by the authors, without undue reservation.
